# Biochemical Effects of the Toxic Interaction of Copper, Lead and Cadmium on Clarias gariepinus

**DOI:** 10.5696/2156-9614-7.16.38

**Published:** 2017-12-18

**Authors:** Olayinka Adunni Ubani-Rex, Joseph Kayode Saliu, Taiwo Hassan Bello

**Affiliations:** Ecotoxicology Unit, Department of Zoology, University of Lagos, Lagos, Nigeria

**Keywords:** sublethal concentration, lipid peroxidation, synergism, antagonistic, heavy metal

## Abstract

**Background.:**

The presence of heavy metals in the aquatic environment is a concern because of potential toxicity and threats to plant and animal life.

**Objective.:**

The present study evaluated the joint action toxicity and biochemical effects of sublethal concentrations of copper (Cu), lead (Pb) and cadmium (Cd) against Clarias gariepinus over a period of 28 days.

**Methods.:**

We procured fingerlings (weight: 5–8 g; length: 4.5–6.0 cm) and juveniles (weight: 20–25 g; length: 14.5–17.5 cm) from a commercial fish farm in Bariga, Lagos state, Nigeria. Test toxicants were selected from the analyzed heavy metals in the field based on their deviation from World Health Organization, Federal Environmental Protection Agency and United States Environmental Protection Agency standards. Fish were randomly loaded into a 4-L glass aquaria for the bioassay per toxicant concentration of two replicates and untreated control (dechlorinated tap water).

**Results.:**

The derived 96 hour lethal concentration 50 (LC_50_) value of Cu (2.11 mg/L) was the most toxic followed by Cd (24.18 mg/L) and Pb (34.48 mg/L), which was the least toxic of the singly tested pollutants. The analysis of dose-response data of the joint action toxicity of Cu and Cd, and Cu and Pb determined 96 hour LC_50_ values of 1.804 mg/L and 2.15 mg/L, respectively. The interactions between the mixture of Cu:Cd conformed with the model of synergism (synergistic ratio (SR)>1 and relative toxic units (RTU)>1), while the interaction between Cu:Pb was found to be antagonistic (SR<1), with an SR value of 0.98. The biochemical effects study revealed that malondialdehyde (MDA) levels decreased significantly (P<0.05) in the exposed fish, reduced glutathione was not significant at (P<0.05), and levels of superoxide dismutase (SOD), catalase, glucose and cholesterol were significantly different (P<0.05).

**Discussion.:**

The observed increased in the glutathione level in the Cu:Cd mixture and a corresponding decrease in MDA concentration in the liver of test animals revealed the ability of fish to overcome the effects of lipid peroxidation in this group because the Cu ion is displaced by Cd, and the fish were able to catalyze the breakdown of hydrogen peroxide via the Fenton reaction.

**Conclusions.:**

Further studies on the joint action toxicity of heavy metals are needed in order to further determine their concentration in the local environment.

**Ethics Approval.:**

Study protocols were approved by the Health Research Ethics Committee of the University of Lagos.

## Introduction

The presence of heavy metals in the aquatic environment is a major concern because of potential toxicity and threats to plant and animal life.^1^ Toxic heavy metals in the aquatic environment can be traced to both natural and anthropogenic sources. Changes arising from anthropogenic activities occur in the aquatic ecosystem, affecting the aquatic habitat and fish.^2^,^3^ Lead and other trace metals have a high affinity for animal tissues where they are concentrated to varying levels.^4–6^ Cadmium (Cd) is a non-essential heavy metal and is considered to be one of the most toxic water contaminants and could cause toxicity in organisms at a number of levels, from populations and communities to cell elements.^7^ Even at sub-lethal concentrations, Cd has a cumulative polluting effect and can cause serious disturbances in fish metabolism such as abnormal behavior, locomotor anomalies or anorexia.^8–10^ Cadmium also affects blood cells.^11^ These toxicants exert their toxic effect by generating reactive oxygen species (ROS), causing oxidative stress. Oxidative stress is an unavoidable aspect of aerobic life. It is the result of an imbalance between the production of ROS and antioxidant defenses in living organisms.^12^ Reactive oxygen species are induced by substances such as transitional metal ions, pesticides, and petroleum pollutants.^13^,^14^ Free radicals are also produced by endogenous cellular sources during normal cell metabolism. Mitochondrial respiration is the main endogenous source of ROS. Elevated production of ROS can cause oxidation of proteins and lipids, alterations in gene expression, and changes in cell redox status.^15^

Mechanisms of antioxidant defenses in fish include the enzyme system and low molecular weight antioxidants, similar to those in mammals, although the specific isoforms of enzymes in various fish species have not been well identified.^16^ Superoxide dismutase, catalase, glutathione peroxidase, and glutathione-s-transferase are the main antioxidant enzymes and important indicators of oxidative stress. Reduced glutathione and oxidized glutathione disulphide play a key role in non-enzymatic antioxidant defense. Metal-binding proteins such as ferritin, ceruloplasmin, and metallothioneins have special functions in the detoxification of toxic metals, and also play a role in the metabolism and homeostasis of essential metals.^17^ Fish are endowed with defensive mechanisms to neutralize the impact of ROS resulting from the metabolism of various chemicals. Exposure of fish to metals may result in increases in ROS, leading to impairment of normal oxidative metabolism and finally to oxidative stress.^14^

The present study assessed the biochemical effects of the toxic interaction of copper (Cu), Cd and lead (Pb) on Clarias gariepinus.

## Methods

Fingerlings (weight: 5–8 g; length: 4.5–6.0 cm) and juveniles (weight: 20–25 g; length: 14.5–17.5 cm) of Clarias gariepinus were procured from a commercial fish farm in Bariga, Lagos state, Nigeria. The procured fish samples were transported to the laboratory in an oxygenated plastic container and kept in 40-litre holding tanks filled with de-chlorinated tap water. During acclimatization, they were fed with 5 percent of their body weight with Coppens fish feed twice daily (morning and evening), and the water was changed every other day to prevent the accumulation of waste metabolite and food particles. They were held in the container for a minimum of 5 days to enable acclimatization to laboratory conditions (temperature: 28 ± 2° C; relative humidity: 78 ± 4%) before the commencement of the experiment. Study protocols were approved by the Health Research Ethics Committee of the University of Lagos.

**Abbreviations**
*LC*_*50*_: Lethal concentration 50
*MDA*: Malondialdehyde
*ROS*: Reactive oxygen species
*RTU*: Relative toxic units
*SOD*: Superoxide dismutase
*SR*: Synergistic ratio
*TBA*: iobarbituric acid
*TCA*: Tricarboxylic acid

Test toxicants were selected from the analyzed heavy metals in the field based on their deviation from World Health Organization, Federal Environmental Protection Agency in Nigeria and United States Environmental Protection Agency standards. Test solutions were prepared by dissolving a set amount of lead dinitrate (Merck, 99.5% purity), cadmium sulfate (HiMedia Lab, 98.5% purity), and copper(II) sulfate pentahydrate (Ajax Chemicals, 98.5% purity) in distilled water producing 100 ppm of stock solution. From these stock solutions, different concentrations of test solutions were prepared through a series of dilutions with de-chlorinated tap water. Range finding was conducted to determine the definitive concentrations to be used for the acute test on both single and joint actions of the toxicants.

Physiochemical characteristics were measured at the beginning of the experiment and at the end (that is, before the change of test media). The parameters measured included dissolved oxygen, total dissolved solids, salinity, conductivity, temperature and pH using a Horiba U-50 multi sampler.

Ten fish were randomly loaded into a 4 L glass aquaria for the bioassay per toxicant concentration of two replicates and untreated control (dechlorinated tap water). The fish were not fed during the 96 hour experimental period. Mortality assessment was carried out once every 24 hour for 4 days. After a range finding test, the test organisms were exposed to prepared concentrations for 96 hour as follows:

Cu—1, 2, 3, 4, 5 and 6 mg/L and untreated control
Cd—5, 10, 20, 30 and 40 mg/L and untreated control
Pb—10, 20, 40, 60 and 100 mg/L and untreated control

A series of bioassays similar to those described for single action tests were carried out, but in this instance, animals of similar sizes were exposed to a binary mixture based on essential and non-essential nutrients (Cu:Cd and Cu:Pb) in a ratio of 4:1 and 2:1, respectively, based on their occurrence in the field.

In this series of experiments, the test animals were exposed to sublethal concentrations (1/10th and 1/100th of 96 hour lethal concentration 50 (LC_50_)) derived from the results of single and joint action toxicity studies of the test compounds and untreated control in replicates for a period of 28 days. A semi-static bioassay test protocol was adopted, in which the test media were changed once every 4 days to fresh media of the same concentration and untreated control. Tested fish were fed during the test periods. At the end of 28 days, fish were sacrificed to obtain liver tissues required for biochemical assays. The liver was removed and washed free of blood in ice cold isolation medium (0.25 M sucrose, 5 mM tris hydrogen chloride (HCL)), lightly blotted and weighed. Samples were then cut into fragments and homogenized (9% w/v) in 100% methanol and centrifuged at 10,000 × g for 15 minutes at 4°C as described by Hermes-Lima M, et al.^18^ The supernatant was collected for substrate and enzyme assays.

The level of homogenized malondialdehyde (MDA), as an index of lipid peroxidation was determined using the method of Buege JA, et al.^19^ Then 1.0 mL of the supernatant was added to 2 mL of (1:1:1 ratio) tricarboxylic acid (TCA)-thiobarbituric acid (TBA)-HCl reagent TBA 0.37%, 0.24N HCl and 15% TCA), TCA-TBA-HCl reagent boiled at 100°C for 15 minutes, and allowed to cool.

Flocculent materials were removed by centrifuging at 3000 rpm for 10 minutes. The supernatant was removed and the absorbance read at 532 nm against a blank. MDA was calculated using the molar attenuation coefficient for MDA TBA complex of 1.56 × 105 M^−1^cm^−1^.

The reduced glutathione content of liver tissue as non-protein sulfhydryls was estimated according to the method described by Sedlak J, et al.^20^ Then 10% TCA was added to the homogenate, centrifuged and 1.0 mL of supernatant was treated with 0.5 mL of Ellman's reagent (19.8 mg of 5, 5-dithiobisnitro benzoic acid in 100 mL of 0.1% sodium nitrate) and 3.0 mL of phosphate buffer (0.2 M, pH 8.0). The absorbance was read at 412 nm.

Superoxide dismutase activity was determined by its ability to inhibit the auto-oxidation of epinephrine determined by the increase in absorbance at 480 nm as described by Sun M, et al.^21^ The reaction mixture (3 mL) contained 2.95 mL, 0.05 M sodium carbonate buffer (pH 10.2, 0.02 mL liver homogenate) and 0.03 mL of epinephrine in 0.005 N HCL was used to initiate the reaction. The reference cuvette contained 2.95 mL buffer, 0.03 mL of substrate (epinephrine) and 0.02 mL of water. Enzyme activity was calculated by measuring the change in absorbance at 480 nm for 5 minutes (∑= 4020 M^−1^cm^−1^). Serum catalase activity was determined according to the method of Beers and Sizer as described by Usoh IF, et al, by measuring the decrease in absorbance at 240 nm due to decomposition in a hydrogen peroxide (H_2_O_2_) UV-recording spectrophotometer.^22^ The reaction mixture (3 mL) contained 0.1 mL of serum in phosphate buffer (50 mM, pH 7.0) and 2.9 mL of 30 mM H_2_O_2_ in a phosphate buffer (pH 7.0). An extinction coefficient at 240 nm H_2_O_2_ of 40.0 M^−1^cm^−1^ was used for the calculation.^23^ The specific activity of catalase was expressed as moles of H_2_O_2_ reduced per minute per mg protein.

Cholesterol content of samples was estimated by the method described by Zlatkis AB, et al.^24^ Next, 0.1 mL each of cholesterol standard and lipid extract was transferred into test tubes and evaporated to dryness in a hot water bath. Then 3 mL of glacial acetic acid was added to each of the test tubes and also a third tube (blank) and shook to dissolve the lipids in the test tubes. Next, 3 mL of color reagent (1 g of ferric chloride dissolved in 10 mL of glacial acetic acid and 1 mL of this solution made up 100 mL with concentrated sulfuric acid) to each of the three test tubes. The solutions were vortexed, allowed to stand and cooled at room temperature for 20 minutes. The absorbance of the standard and lipid extract was measured against the blank at 560 nm in a spectrophotometer. The glucose content was estimated by the glucose oxidase method.^25^

Toxicological data involving quantal response (mortality) for both single and joint action studies were analyzed by probit analysis including the equation for probit lines.^26^ This was executed using the Statistical Package for the Social Sciences program for Windows (SPSS 16.0). The indices of toxicity measurement derived from these analyses were LC_50_ (lethal concentration causing 50% response (mortality) of the exposed organisms), toxicity factor (TF) and 95% confidence limits employed as follows:

For the joint action toxicity of the heavy metal mixtures, the two models employed for the classifications were the concentration-addition model described by Anderson PD, et al. with slight modification [relative toxic units (RTU) estimations] and the synergistic ratio (SR) model.^27–29^

### Model 1—Concentration-Addition Model:

(i) Additive if observed LC_50_ value of the mixture is equal to the predicted LC_50_ value i.e. RTU = 1,
(ii) Synergistic if the observed value of the mixture is less than the predicted LC_50_ value i.e. RTU > 1,
(iii) Antagonistic if the observed LC_50_ value of the mixture is greater than the predicted LC_50_ value i.e. RTU < 1

The relationship of derived LC_50_ values to predicted LC_50_ (RTU) is estimated as:

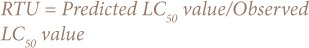



### Model 2—Synergistic Ratio (SR) Model:


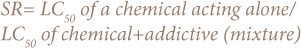

Where SR =1 joint action is described as additive
SR<1 joint action is described as antagonistic
SR> 1 joint action is described as synergistic

The lipid peroxidation and enzyme activity measurement data were subjected to one-way analysis of variance between the different treatment means and the control. Significant difference was determined at a 5% confidence level (P<0.05) using GraphPad Prism 5.

## Results

The analysis of the physiochemical parameters of the test media showed that the dissolved oxygen level ranged from 3.96 mg/L (after 4 days of exposure) to 6.2 mg/L (after each change to a clean media). The pH and salinity of the test media ranged from 9.02–6.9 and 0.1–0.05 ppt, respectively. The conductivity and total dissolved solids in the test media increased from 0.195 to 0.298 mS/cm and 0.98 to 0.127 g/L, respectively, over the study period.

On the basis of the derived 96 hour LC_50_ values (*Table 1*), Cu was the most toxic pollutant (2.11 mg/L), followed by Cd (24.18 mg/L) and Pb (34.48 mg/L). The computed toxicity factor revealed that Cu is approximately 12× and 16× more toxic than Cd and Pb, respectively.

**Table 1 —:**
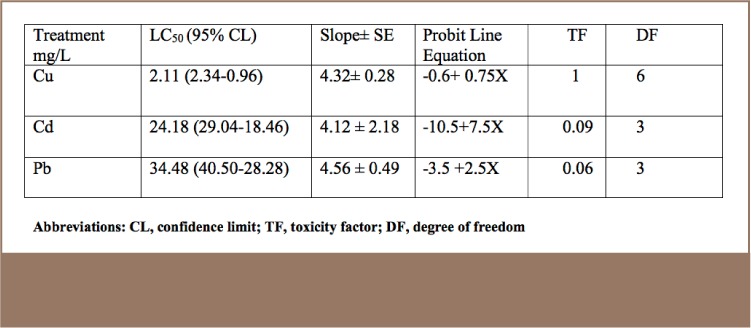
Single Action Toxicity of Cu, Cd and Pb Against Clarias gariepinus Fingerlings Based on 96 Hour Mortality Data

The analysis of dose-response data of the joint action toxicity of Cu with other metals (Cd or Pb) is shown in Table 2. The 96 hour LC_50_ values of Cu:Cd and Cu:Pb were 1.804 ppm and 2.15 ppm, respectively. The interactions between the mixture of Cu:Cd conformed with the model of synergism (SR>1 and RTU>1).^27^,^28^ The joint action of the toxicants on the tested organisms makes the compound highly toxic. On the other hand, the interaction between Cu:Pb was found to be antagonistic (SR<1), with an SR value of 0.98.

**Table 2 —:**
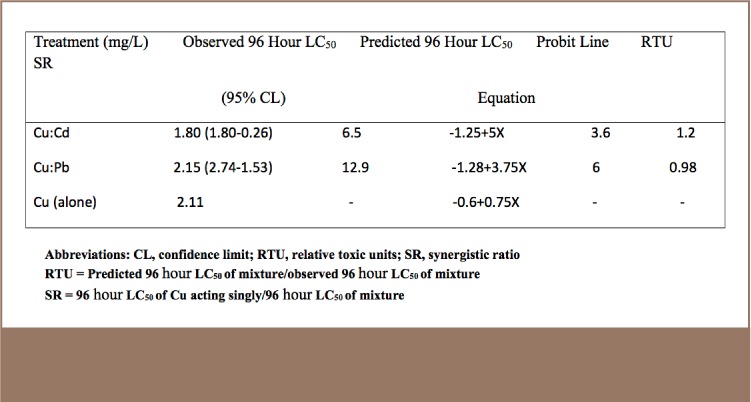
Analysis of 96 Hour LC_50_ Values of Cu and Cd (Ratio 4:1) and Cu and Pb (Ratio 2:1) Against Clarias gariepinus Juveniles

**Figure 1 —:**
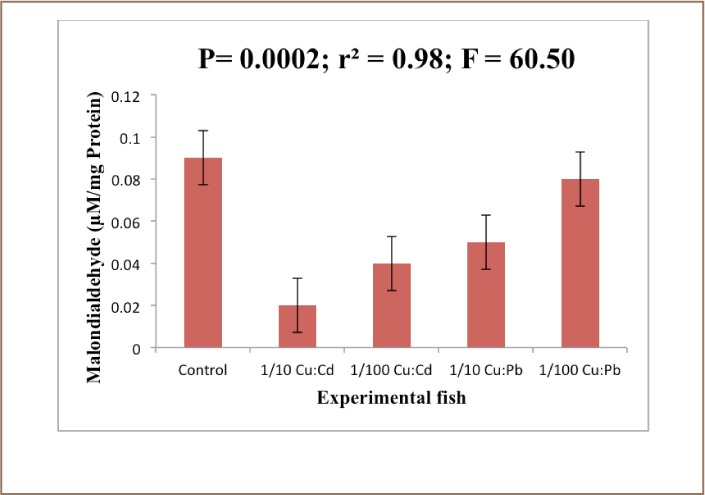
Malondialdehyde levels in the liver of Clarias gariepinus exposed to Cu: Cd (ratio 4:1) and Cu:Pb (ratio 2:1) over a period of 28 days. Values represent mean ± standard error (n=10 per treatment). Mean significantly different at (P<0.05)

**Figure 2 —:**
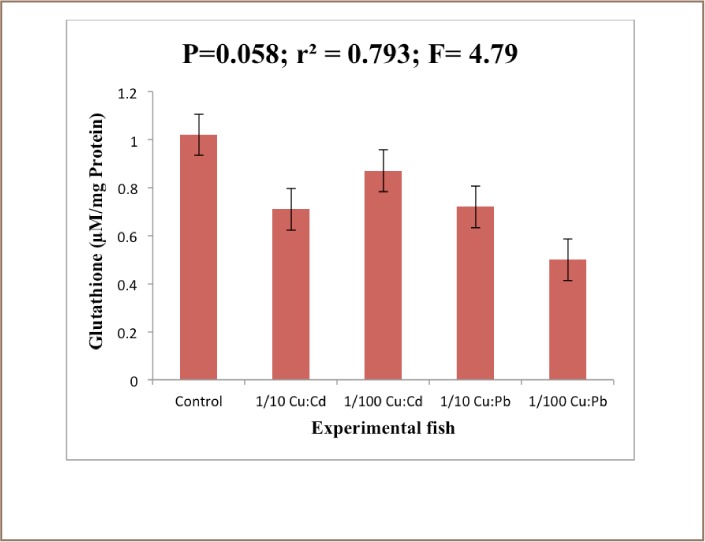
Glutathione levels in the liver of Clarias gariepinus exposed to Cu: Cd (ratio 4:1) and Cu:Pb (ratio 2:1) over a period of 28 days. Values represent mean ± standard error (n=10 per treatment). Mean difference not significant at (P<0.05)

**Figure 3 —:**
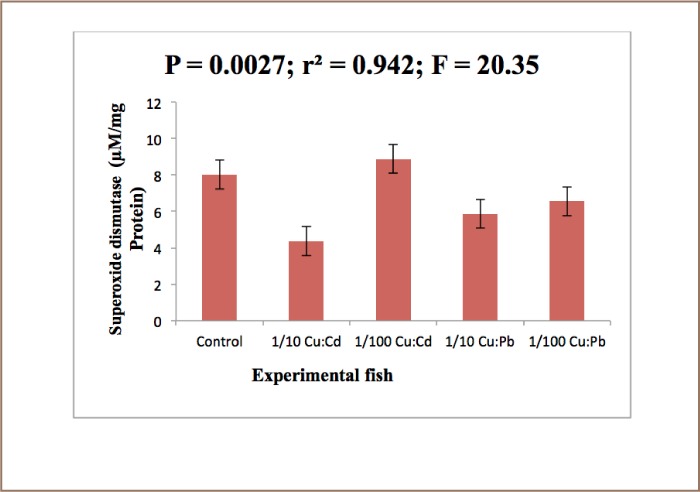
Superoxide dismutase levels in the liver of Clarias gariepinus exposed to Cu:Cd (ratio 4:1) and Cu:Pb (ratio 2:1) over a period of 28 days. Values represent mean ± standard error (n=10 per treatment). Mean difference significant at (P<0.05)

The results revealed that the MDA level in the liver of the exposed fish decreased significantly (P<0.05) compared to the control animals after 28 days of exposure (*Figure 1*). There was a reduction in the levels of glutathione in the treated groups compared to the control. There was no significant difference (P<0.05). Figure 3 showed that there was a significant difference (P<0.05) in the superoxide dismutase (SOD) level of exposed fish over 28 days (*Figure 2*).

**Figure 4 —:**
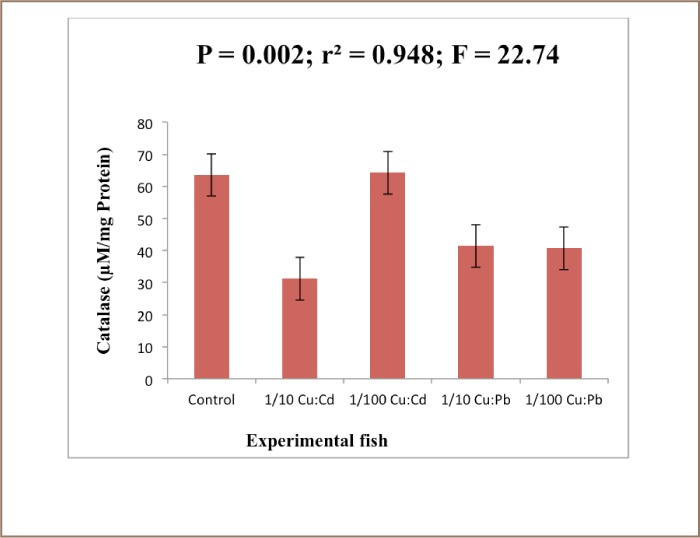
Catalase levels in the liver of Clarias gariepinus exposed to Cu: Cd (ratio 4:1) and Cu:Pb (ratio 2:1) over a period of 28 days. Values represent mean ± standard error (n=10 per treatment). Mean difference significant at (P<0.05)

**Figure 5 —:**
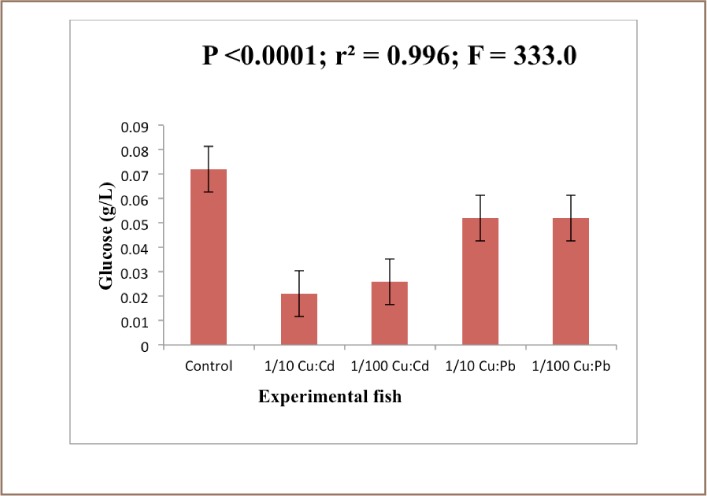
Glucose levels in the liver of Clarias gariepinus exposed to Cu: Cd (ratio 4:1) and Cu:Pb (ratio 2:1) over a period of 28 days. Values represent mean ± standard error (n=10 per treatment). Mean difference significant at (P<0.05)

**Figure 6 —:**
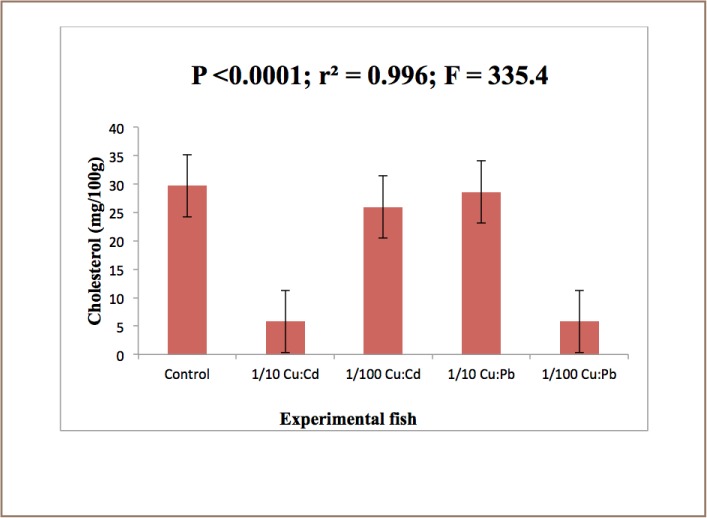
Cholesterol levels in the liver of Clarias gariepinus exposed to Cu: Cd (ratio 4:1) and Cu:Pb (ratio 2:1) over a period of 28 days. Values represent mean ± standard error (n=10 per treatment). Mean difference significant at (P<0.05)

SOD is known to provide cytoprotection against free radical-induced damage by converting superoxide radicals generated in peroxisomes and mitochondria to hydrogen peroxide. The result further showed that there was a significant difference (P<0.05) in catalase activity between exposed and control animals (*Figure 4*). The mean difference in levels of glucose and cholesterol in the control and exposed liver was significant at (P<0.05) (*Figures 5 and 6*).

## Discussion

This present study investigated the individual and joint action of three toxic metals (Cu, Pb and Cd) frequently existing in contaminated areas as an oxidative stressor in Clarias gariepinus. The LC_50_ value of Pb was similar to the data for Clarias gariepinus found in numerous studies as well as similar data for Clarias angularis for Cu.^30–32^ The computed toxicity factor revealed that Cu is approximately 12× and 16× more toxic than Cd and Pb, respectively. However, this conforms to the findings of Claire CL, et al. on the toxicity of Cu, Cd and Pb metal ions in Chironmids and factors that affect metal accumulation.^33^ This result also corroborates the report of Fafioye O, et al. for Penaeus monodon exposed to Cd, Cu and Pb, and contradicts the findings of Otitoloju AA, et al. that Cd was more toxic than Cu, Zn and Pb when tested against lagoon animals such as Clibanarius africanus, Tympanotonus fuscatus and Sersema spp. Oyewo EO had a similar observation and recorded differential toxicities of heavy metals against different test organisms.^34–36^

The differential toxicities observed among the sampled toxicants can be attributed to individual persistence and penetrability of the toxicants into living organisms, site of metabolism action and hence the toxic actions they exert on the exposed organisms.

The analysis of dose-response data of the joint action toxicity of Cu with other metals (Cd or Pb) found that the interactions between the mixture of Cu:Cd conformed with the model of synergism.^28^,^29^ The joint action of the toxicants on the tested organisms makes the compound highly toxic. The implication of this is that in terms of toxicity, any compound or pollutant containing both Cu and Cd should be carefully injected into the system as it enhances bio-toxicity. On the other hand, the interaction between Cu:Pb was found to be antagonistic. This finding is similar to the result of Xu X, et al. using sea urchin embryo larvae.^37^ Consequently, the mixture was less toxic than Cu when acting singly against the test organism. This implies that this compound has reduced toxicity on life forms in the environment, which might be explained by the fact that Cu could decrease the absorption of the mixture, thus reducing its toxicity.^38^

The heavy metals Pb, Cu and Cd, all have electron-sharing affinities that can result in the formation of covalent attachments mainly between heavy metals and sulphydryl groups of proteins. The result indicates significant lipid peroxidation in the liver of the tested fish.

The relationship between exposure to toxicants and enzymes such as catalase, superoxide dismutase, reduced glutathione, and glutathione-S-transferase, as well as lipid peroxidation products have been the subject of several investigations. Furthermore, the activities of SOD, MDA, catalase and the redox sensitive thiol compound glutathione were elevated in the species liver. The significant increase in this organ may be a response to oxidative stress caused by the presence of heavy metals. The accumulation of heavy metals might have led to the production of superoxide anions which led to the induction of SOD to convert the superoxide radical to H_2_O_2_.

An increase in the activity of catalase and SOD is usually observed in the face of environmental pollutants since the SOD-catalase system represents the first line of defense against oxidative stress.^39^ However, in the present study, the activity of MDA was decreased in the liver of all of the tested species. Hence, a decrease in liver MDA levels is an indication of a lower level of lipid peroxidation.^40^ This decrease in MDA is consistent with the finding of Saliu JK, et al. who reported a reduction in MDA levels in fish exposed to sublethal concentrations of lead salts (Pb(N0_3_)_2_) and also that of Sogbamu TO, et al. who reported a reduction in the levels of MDA in fish exposed to dispersant mixtures over a period of 28 days and contradict the findings of Arafa MM, et al. who reported that malondialdehyde was significantly increased in the liver of Clarias gariepinus exposed to heavy metal pollution.^41–43^ The significant reduction in the concentration of the MDA in the treated groups could be attributed to the action of the antioxidant enzymes in preventing cellular injury by ROS. Lead has been reported to have no pro-oxidant catalytic activity with respect to lipid peroxidation (LPO). Yiin SJ, et al.^44^ demonstrated a significant enhancement of MDA when Pb was incubated with linoic, linolenic and arachidonic acid. Several studies have shown that Pb alters the activity of antioxidant enzymes like SOD, catalase, glutathione peroxidase and glucose 6-phosphate dehydrogenase and antioxidant molecules like glutathione in animals and humans.^45–48^

Watanabe M, et al. showed generation of non-radical hydrogen peroxide which by itself became a significant source of free radicals via the Fenton chemistry.^49^ It has also been suggested that Cd could induce oxidative damage by causing the intercellular accumulation of ROS such as superoxide radicals O_2_− and nobelium, thus increase the sensitivity of cell to toxicants.^50–52^

Cadmium could replace iron and Cu from a number of cytoplasmic and membrane proteins like ferritin, which in turn would release and increase the concentration of unbound iron or Cu ions. These free ions participate in causing oxidative stress via the Fenton reaction.^53^,^54^ Recently, Watjen W, et al. showed evidence in support of the proposed mechanism.^55^ They found that Cu and iron ions displaced by Cd were able to catalyze the breakdown of hydrogen peroxide via the Fenton reaction.^54^

Casalino E, et al. proposed that Cd binds to the imidazole group of histone H3.3-like type 2 in SOD which is vital for the breakdown of hydrogen peroxide, thus causing its toxic effects.^53^ Cadmium inhibition of liver mitochondrial antioxidant manganese superoxide dismutase activity was completely removed by manganese(II) oxide ions, suggesting that the reduced effectiveness of this enzyme is probably due to the substitution of Cd for manganese. Gravato C, et al. attribute the depletion of glutathione to direct Cu interference with glutathione synthesis, inhibition of glutathione reductase, and the participation of glutathione as a substrate in detoxification reactions.^56^

The depletion of glutathione in fish muscle after copper sulphate exposure has also been reported by Jena SD, et al.^57^ Ahmad I, et al. reported that metallothionein induction plays a role in the oxidative defence against chronic Cu exposure in the liver of a freshwater catfish, Channa punctatus.^58^

Acute intoxication of animals with Cd has been shown to cause increased activity of antioxidant defense enzymes like Cu-zinc containing superoxide dismutase, catalase, glutathione peroxidase, glutathione reductase and glutathione-S-transferase.^59^

Apart from oxidative stress-mediated toxicity, Cd is also known to cause deleterious effects by deactivating DNA repair activity.^60^ The decrease in the cholesterol and glucose level in Clarias gariepinus exposed to the heavy metals contradicts the findings of Nath K, et al. and James R, et al. who reported that blood glucose and cholesterol levels rise in response to toxicants.^61^,^62^

## Conclusions

The use of biochemical responses in heavy metal biomonitoring in fish is very effective as a biomarker of pollution incidents, both in the field and the laboratory. The results from the present study show that the oxidative stress response can serve as a useful tool in environmental monitoring. However, there is a need for further research on joint action toxicity of the mixture of heavy metals and continuous biomontoring of heavy metals pollution in the field.
